# Exploring the Intention to Adopt Sustainable Mobility Modes of Transport among Young University Students

**DOI:** 10.3390/ijerph20043196

**Published:** 2023-02-11

**Authors:** Carlos J. Rodríguez-Rad, María-Ángeles Revilla-Camacho, María-Elena Sánchez-del-Río-Vázquez

**Affiliations:** Department of Business Administration and Marketing, Universidad de Sevilla, 41004 Sevilla, Spain

**Keywords:** mobility, sustainability, diffusion, adoption, market-based management, market orientation, customer perception, PLS

## Abstract

This research arises from the need to accelerate the diffusion of sustainable mobility and the call for research on this topic. The Sustainable Development Goal 11 of the 2030 Agenda, the scientific articles on sustainable mobility systems and the advance of micro-mobility, shared mobility, Mobility on Demand (MOD) or Mobility as a Service (MaaS) in recent years manifest the importance of sustainable urban development. Given this reality, this paper explores the elements and factors that condition the adoption of a sustainable mobility mode of transport. An empirical study was carried out in the city of Seville through an electronic questionnaire delivered to university students. Our exploratory approach is an innovative point of view that can help us to better understand the reasons for the successful adoption of sustainable mobility modes of transport. The most relevant results of this study indicate that the user’s perceived impacts on sustainability and customer forces influence the adoption of a mode of transport by citizens, whereas the product forces seem not to influence thTABLEis. Therefore, cities and companies that have put their emphasis exclusively on improving mobility products and services, without taking citizens into account, are less likely to succeed. Additionally, governments should consider that economic issues or environmental concerns of citizens act as drivers of innovation in urban mobility.

## 1. Introduction

In 2015, the United Nations (UN) approved the 2030 Agenda for Sustainable Development through 17 Sustainable Development Goals (SDGs). The 2030 Agenda becomes an opportunity for countries and their societies to embark on a new path to improve the lives of all. The world is increasingly urbanized, already generating 70% of the world’s carbon emissions and more than 60% of resource use. By 2030, 60% of the population is expected to live in cities. This gives rise to SDG11, which refers to the transition to more inclusive, safe, resilient, and sustainable cities. SDG11 has come to guide the management of cities around the world in recent years; consequently, nations are actively trying to rebuild their political policies to improve the sustainability of society [1,2].

Among the drivers of innovation in urban mobility, therefore, in addition to demographic and lifestyle changes, economic issues or environmental concerns of citizens [3], we find sustainability and public policies and legislation, as well as technological changes [4]. New urban mobility solutions should contribute to greater sustainability, quieter and less polluted cities [5]. It is not surprising, therefore, that smart mobility projects are the first step towards a Smart City [6]. On the other hand, the proliferation of scientific articles on sustainable mobility systems in recent years reveals the urgency of authorities to prioritize sustainable urban development [7]. In this regard, sustainable mobility solutions are becoming increasingly necessary to achieve transport services with less impact on the environment, with lower emissions of polluting gases, reduced noise and urban development that favors quality of life in general [8,9].

There is a clear need to accelerate the diffusion of sustainable mobility technologies and, consequently, to focus attention on assessing how to accelerate transitions towards more sustainable production and consumption systems. However, the acceleration of this diffusion has not been studied in detail [5]. In this line, Munkácsy and Monzón [4] support that although research on individual travel decision-making (choice of mode, route, etc.) is extensive, the question of how travelers or users of a transport service perceive the characteristics of an innovation and how they react requires further study. In addition, Min et al. [10] indicate that in the field of the sharing economy as a whole, most recent studies have focused on the business or government perspective, its impact on the tourism sector, regulation, or its impact on overall sustainability, rather than focusing on the consumer perspective. Specifically, in the field of mobility, Ahn and Park [2] and Wang et al. [11] state that few studies so far have focused on citizens’ perceptions of sustainable transport systems, which are widespread in both developed and developing countries, and therefore advocate the need to carefully consider the user’s perspective in the development of mobility initiatives.

In this sense, this study aims to fill this gap in the literature, focusing on the consumer´s perception of the factors that influence the speed of diffusion of mobility modes of transport offered in the city of Seville and its metropolitan area. Another great contribution of this research is to differentiate between product forces and customer forces and their relationship with the intention of use. To facilitate success, market orientation suggests that the analysis of consumer perceptions should be a priority when designing and launching an innovation, beyond the analysis of product features. On the other hand, since the Brundtland Commission in 1987, scholars and policy professionals have focused great concern on sustainable development that must meet the needs of the present without sacrificing the ability of future generations to do the same, namely economic, social and human development, and human, environmental and ecological health [12]. However, studies on consumers’ perception of the ecological, social, and economic impacts of mobility solutions and their influence on their adoption are very scarce and limited [13]. Consequently, this paper also strives to fulfill this gap.

The adoption rate (i.e., the relative speed with which an innovation is adopted) can be explained by differences in perception of certain attributes of an innovation [4]. It is the acceptance and continued use by individuals of a new idea or thing that generates its diffusion [2]. The innovation diffusion theory (IDT), developed by Roger [14], was born to explain why individuals adopt or reject an innovation based on their beliefs. Roger focuses these beliefs on five attributes of innovation, which are relative advantage, compatibility, complexity, trialability and observability. Innovations will be accepted more quickly if they have characteristics that distinguish themselves from others, are compatible with users’ values, are easy to use, can be tested by users, and are visible. Roger [14] is one of the most cited references in the literature about innovation diffusion [4,15]. However, its characteristics do not fully measure the variety of perceptions of existing innovations [14]; consequently, Roger’s model has been extended in subsequent research. In this line, Moore [16] propose the model of the perceived characteristic of innovation (PCI) to examine the perception of possible adopters about the use of an innovation. The authors identified eight PCIs: relative advantage, ease of use, compatibility, image, result demonstrability, visibility, voluntariness and trialability. The image refers to the degree to which its use is perceived to enhance the user’s image or status and Roger [14] includes it in relative advantage. These authors also add voluntariness, which is the degree to which the use of the innovation is perceived as voluntary. Rogers’ observability was further divided into demonstrability and visibility.

Technological development and the growing concern on sustainability, as mentioned above, have driven research on mobility modes of transport. Not surprisingly, some of these studies are based on IDT. Furthermore, some of these investigations have chosen to combine IDT with other models to examine the factors that influence the intention to use different mobility modes of transport. These other models include Technology Acceptance Model (TAM) by Davis [17], Theory of Planned Behavior (TPB) by Ajzen [18] or Unified Theory of Acceptance and Use of Technology (UTAUT) by Venkatesh et al. [19].

Among sustainable mobility studies, Munkácsy and Monzón [4] and Wang et al. [20] focus on IDT to study the attributes influencing users’ adoption of the public bicycle system (i.e., shared bicycle systems) in Madrid (Spain) and Beijing (China), respectively. Verma et al. [21] rely on IDT to explain the electric vehicle (EV) use in Bengaluru (India). On the other hand, Ahn and Park [2] base their research on IDT and TAM to explain the intention to use sustainable transportation in South Korea. In the field of autonomous vehicles (AV), Yuen et al. [22] also use both models in a survey conducted in Beijing (China). In the field of collaborative transport, Min et al. [10] integrate IDT and TAM to discover the factors of Uber mobile app adoption among users. Again, in the case of shared bicycles, Chen and Lu [1] combine IDT and TAM in their study in Taipei (China). In the case of automated shuttles in Berlin’s public transport system, Nordhoff et al. [23] chose to integrate IDT and UTAUT. The combination of IDT and TBP is the basis of Eccarius and Lu’s [24] research on the adoption factors of an electric scooter sharing service among university students in Taiwan.

The rationale for extending the models is to improve their predictive power, so, like IDT, the other models are sometimes used according to extended versions. Therefore, under the innovation diffusion theory (IDT) umbrella, this study bases its research on Best [25,26] and Hawkins and Mothersbaugh [27] to propose forces to explain the speed in the adoption of an innovation and thus its diffusion. According to Best [25], our model distinguishes between product forces and customer forces. In addition, it was decided to incorporate the perception of the impacts of mobility on the sustainability of cities, from its triple ecological, economic and social dimension, in order to comply with the complete concept of sustainability. All of this was done with the aim of better explaining the adoption of sustainable mobility modes of transport.

To achieve the proposed aims, the partial least squares structural equation modeling (PLS-SEM) technique is used on data collected from university students in the city of Seville. The city of Seville is a southern European city that is making important efforts to improve mobility and has developed a Sustainable Urban Mobility Plan (SUMP) to achieve a more sustainable city by adopting a mobility model that is more efficient from an economic and functional point of view, more equitable from a social point of view and more protective from an environmental point of view [28]. The most relevant results of our study indicate that impacts on sustainability and customer forces have a strong influence on the adoption of a mode of transport by citizens, whereas the product forces seem not to have a strong influence on the adoption of a more sustainable mode of transport. The city of Seville is also an exponent of multimodality, offering citizens and tourists a wide variety of transport alternatives available: moped sharing, e-scooter sharing, e-bike sharing, bike sharing, taxi, VTC (i.e., ride-sourcing), bus, metro, tram, train, etc.

Seville is the fourth most populated city in Spain, after Madrid, Barcelona and Valencia, with almost seven hundred thousand inhabitants. Seville stands out in terms of vehicle density (6.8 vehicles per 10 inhabitants), length of cycling lanes (189 km) and public transport fleet, with subsidized fares for the elderly or people with functional diversity (disability). It also has relatively low greenhouse gas (GHG) emissions (2.8 tons of CO_2_ equivalent per inhabitant per year) compared to other Spanish cities [28].

Seville’s sustainable urban mobility also has deficiencies, although the development of the SUMP is an indication of the willingness of those in power to comply with the SDGs 2030. In Seville, transport is the main GHG emitter (45% of total emissions) and cars, which represent 40% of the modal distribution of residents’ trips, generate the most GHGs. This use of private vehicles may be due to some shortcomings in infrastructure and public transport. Among these deficiencies, we can highlight the poor coverage and inadequate intervals of the metropolitan train network during rush hour, the reduced accessibility of train stations for people with disabilities, the unreliability and low commercial speed of the metropolitan bus, the disconnected network of bus lanes at some points of urban public transport (TUSSAM) and its consequent excessive travel time, a single metro line, the lack of continuity of the network of cycle lines in some areas of industrial and commercial estates, the scarcity of bicycle parking spaces and their poor safety and convenience, the high weight of public bicycles, the existence of points of conflict with pedestrians, a deficient system of affordable transport fares for all sections of the population, etc. [28].

In an attempt to overcome these deficiencies, SUMP proposes the following general objectives: mobility as a citizen’s right, guaranteeing trips by a mode of transport with an average of 20 min, the reduction of emissions in Seville by 55% and the integration of the sustainable mobility model of the metropolitan area. Therefore, it is committed to reducing the use of the car, to ensure that 2/3 of trips are made in a sustainable way (pedestrian, bicycle and public transport). It also aims to ensure that 75% of public transport journeys are made in zero-emission vehicles and to establish integration elements to improve the transport system between Seville and the metropolitan area.

Considering, in addition, the reality that 40% of young people between 18 and 25 years of age choose not to get their driving license, making motorcycles, bicycles and electric scooters an alternative for a growing number of citizens, the SUMP plans different lines of action that have already borne fruit. Therefore, Seville has expanded the TUSSAM fleet and has continued with its decarbonization process using compressed natural gas, has launched rental services for electric bicycles, electric scooters and electric motorcycles, has improved and expanded the network of electric scooters and electric bikes, has expanded the bicycle parking facilities in metro stations, has improved and expanded the bicycle lane network, has announced the start of work on the new metro line in 2023, has established Zones 30 and Zones 20 that limit speed to 30 or 20 km/h, respectively, on certain streets to safeguard bicycle or scooter users and pedestrians, has begun work on the extension of the tram line that will link the city’s main intermodal nodes (i.e., Prado de San Sebastián, San Bernardo and Santa Justa), has legislated in search of a better coexistence of cab and VTC, has offered discounted tariffs on trains and buses for different sections of the population, has established low emission zones with restricted access to vehicles with environmental badge type A (i.e., highly polluting), has pedestrianized more streets, has widened sidewalks in some streets, etc.

While the governors demonstrate their commitment to sustainable mobility through this SUMP, they also recognize that its success requires a public–private partnership and citizen participation [28]. Moreover, as urban planning shifts from car-oriented to people-centered [29], the role of the citizen in the adoption of more efficient, environmentally friendly and shared modes of mobility becomes decisive.

The paper is divided into five sections. Following this introduction, the second section presents the material, and the method that explains the model proposed and the method employed to answer our research objectives. The remaining sections show the results obtained with the empirical testing, as well as a detailed discussion of the results, the limitations of our study, and our proposal for future research lines. The paper ends with the conclusions drawn from our study.

## 2. Materials and Methods

The present research analyzes customer perception of the factors that influence the diffusion of sustainable mobility modes of transport and the relationship between these factors and the user’s intention to adopt a sustainable mobility mode of transport. These factors are customer adoption forces, product adoption forces and the impacts of mobility on the sustainability of the city. This study also extends the IDT, as it is based on the factors proposed by Best [25,26] and Hawkins and Mothersbaugh [27] that affect the speed of diffusion of innovations. Hawkins and Mothersbaugh [27] state that diffusion rate of an innovation depends on the customer type of group, type of decision, marketing effort, fulfillment of felt need, compatibility, relative advantage, complexity, observability, trialability and perceived risk. For his part, Best [26] further suggests distinguishing between product forces and customer forces. The latter aspect is fundamental in highly market-oriented companies, as it reinforces the concern for knowing customers’ perceptions on product-related aspects and on customer-related aspects regarding innovation. Customer forces include need, risk, buying decision, observability and trialability; product forces include benefit advantage, affordability, ease of use, desired performance and availability. Best [25] also adds references within customer strengths and services within product strengths.

Based on the fact that the speed at which a new product is adopted by the mainstream market also depends on the characteristics of the customers, not just the characteristics of the product, Best [25,26] argues that the market-oriented strategies needed to accelerate market growth should address both customer adoption forces and product adoption forces. Customer forces refer to those innovation-related customer characteristics that affect the rate at which customers enter a market. Product forces refer to the strength of a product’s positioning that influences the growth rate of the market. Our proposed model includes six customer forces: ease of purchase, trialability, observability, references, avoid risk, felt need. On the other hand, six product forces are included: affordability, product advantages, simplicity of use, availability, services and risk of non-compliance. The description of these forces is shown in Table 1.

On the other hand, the concept of sustainability comes from the environmental field, but it has been applied to different areas and there seems to be consensus on the comprehensive study of sustainability encompassing social, economic and environmental aspects [30,31]. This approach is clearly displayed in Deakin’s [32] definition of sustainable transport in which he states that it is “transportation that meets mobility needs while also preserving and enhancing human and ecosystem health, economic progress, and social justice now and for the future” (p. 6).

Hence, many sustainable mobility researchers advocate its threefold ecological, social and economic dimensions (e.g., [2,12,33,34]). However, only few studies focus on its ecological branch via ecological concern or knowledge (e.g., [1,20,21,35]). Additionally, even scarcer are those that have investigated consumers’ perception of the ecological, social and economic impacts of mobility transport modes and their influence on consumers’ intention to use transport modes [13].

We therefore agree with Bąk et al. [13] on two issues. On the one hand, that sustainable development is one of the greatest contemporary challenges and mobility can offer great opportunities, and on the other hand, that the impact of mobility on the sustainability of cities should be analyzed in terms of three dimensions: economic (e.g., additional income and savings), social (access to services and comfort) and environmental (reduction in energy consumption, greenhouse gas emissions, traffic congestion and noise). Furthermore, given the importance of market orientation, the users’ perception of the city’s sustainability impacts should be analyzed more specifically to assess its influence on the adoption of different modes of transport.

Consequently, perceived impacts on the sustainability of the city (PI) refer to the user’s impression of the effects of a transport mode on the city’s economy, society and environment, i.e., on its sustainability.

User intention (UI) is the subjective likelihood that an individual will engage in a certain behavior [36]. Therefore, it is considered an attitudinal construct that acts as a trigger for making a decision to perform or not perform a behavior, as it denotes the purpose of carrying out a specific behavior.

According to these theoretical bases, we propose the following hypotheses:

**H1.**  *Customer factors are positively related to user intention to adopt a sustainable mobility mode of transport*.

**H2.**  *Product factors are positively related to user intention to adopt a sustainable mobility mode of transport*.

**H3.**  *High positive perceived impacts on the city’s sustainability are related to user intention to adopt a sustainable mobility mode of transport*.

To test the hypotheses proposed, an empirical study was conducted, based on an electronic questionnaire given to university students in the city of Seville aged 18 years old and over. The selection of the respondents was performed with a convenience sample, using emails and social networks, obtaining a total of 210 valid questionnaires after eliminating those that were incomplete or contained systematic or inconsistent answers, as well as observations that did not fulfill the criteria suggested by Hair et al. [37] to handle lost data. The minimum sample size requirement needed to detect minimum R^2^ values of 0.1 in any of the endogenous constructs of the structural model, for 1% significance levels, assuming a statistical power level of 80%, is 145 observations [37]. The collection of information via the online questionnaire was carried out during the month of November 2022. The questionnaire was divided into four sections: the first collected general information from the respondent; the second measured the users’ intention to adopt a sustainable mobility solution; the third collected information about the customer and product adoption forces; and the fourth was related to the users’ perception of the impacts of the solution on urban sustainability. The questions are listed in Table 2. The data analysis used the IBM SPSS 26 [38] and SmartPLS 4 [39] statistical programs. Partial least squares structural equation modeling (PLS-SEM) is a method for analyzing complex interrelationships between constructs and indicators [40]. Compared to other methods, PLS-SEM offers much more flexibility in terms of modeling and data requirements. The technique “thereby overcomes the apparent dichotomy between explanation—as typically emphasized in academic research—and prediction, which is the basis for developing managerial implications” [37] (p. 3). PLS-SEM results are analyzed following a systematic process. The evaluation of the quality of the measurement and structural models focuses on statistics that indicate the predictive ability of the model. In this way, the following evaluation statistics are available: (1) reflective measurement models: internal consistency (coefficient alpha, rho_A or composite reliability), convergent validity (external loadings and average variance extracted, AVE) and discriminant validity (Fornell-Lacker criterion or heterotrait–monotrait ratio, HTMT); (2) formative measurement models: collinearity between indicators (variance inflation factor, VIF) and the magnitude and significance of indicator weights; (3) structural model: magnitude and significance of path coefficients, coefficient of determination (R^2^), redundancy index, goodness-of-fit statistics and predictive relevance. The main reasons for choosing PLS-SEM are the following [37]: on the one hand, the study focuses on the prediction of the dependent variable; on the other hand, the work employs a formative measurement model. Finally, the PLS-SEM characteristic of higher statistical power is quite useful for exploratory research which examines a less developed or still developing theory [41]. The description of the sample is detailed in Table 3.

Regarding the measurement instruments used, all the constructs were measured based on 5-point Likert scales where value five indicates the best situation possible. All the scales are made up of indicators extracted from the theoretical review carried out.

Product factors (PF)—to measure this multidimensional construct, we used a scale with nineteen indicators, following the dimensions proposed by Best [25,26] and Hawkins and Mothersbaugh [27], and specifically adapted to our study. This scale considers PF to be a multidimensional construct, made up of six dimensions: affordability, simplicity of use, availability, product advantages, service and risk of non-compliance. These dimensions do not need to co-vary to describe the product as a factor of adoption. In this case, the idea that PF dimensions tend to vary independently instead of co-varying endows them with the character of formative constructs.

Customer factors (CF)—to measure this multidimensional construct, we used a scale with nineteen indicators, following the dimensions proposed by Best [25,26] and Hawkins and Mothersbaugh [27], and specifically adapted to our study. This scale considers CF to be a multidimensional construct, made up of six dimensions: ease of purchase, trialability, observability, references, avoid risk and felt need. These dimensions do not need to co-vary to describe the product as a factor of adoption. In this case, the idea that CF dimensions tend to vary independently instead of co-varying endows them with the character of formative constructs.

Perceived impacts on the city (PI)—this construct was measured with a four-item scale, related to the perceptions of environmental, economic, social and global impacts of sustainable mobility modes of transport on the future development of the city. The scale comes to indicate, therefore, the impact of the mode of transport on the sustainability of the city.

User intention (UI)—to measure the user intentions, we use a scale of four indicators, adapted from Ray and Sahney [42]. As can be seen in Table 2, the scale contemplates both the intention to use a sustainable mode of transport and the intention to recommend it to third parties. This intention to recommend is a first-level variable, since word-of-mouth is one of the best ways of acquiring new customers.

**Figure 1:**
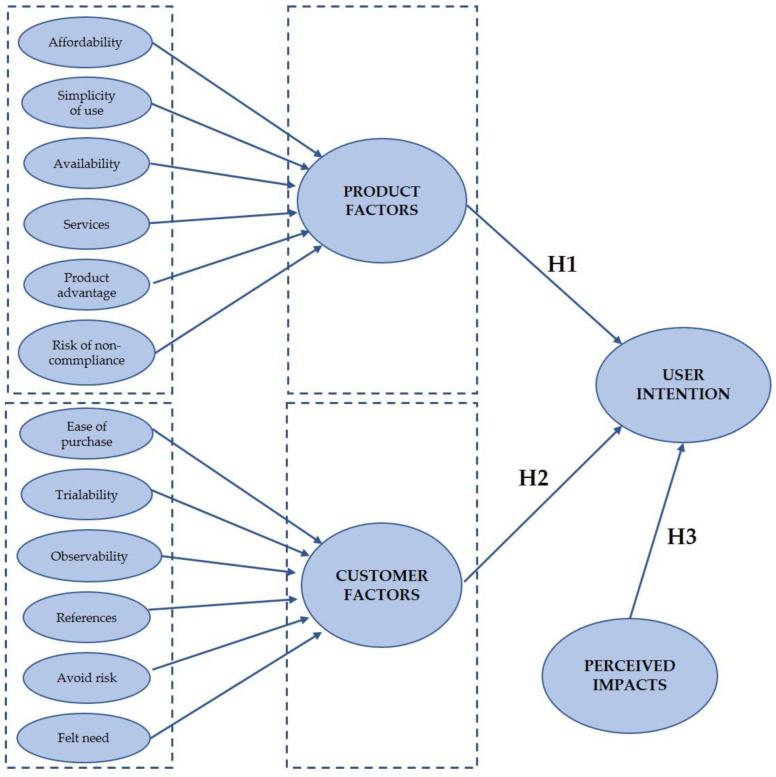
Conceptual framework.

The model proposed is shown in Figure 1.

## 3. Results

Data analysis begins with the valuation of the first-order measurement model. This leads to the refining of the scales with reflective indicators. The evaluation of the measurement model involves the analysis of the item’s individual reliability (loadings), the internal consistency or scale reliability (Cronbach alpha and composite reliability), its convergent validity (AVE) and its discriminant validity (HTMT). Table 3 and Table 4 show the values of all these measurements for the first-order model, entirely made up of reflective indicators.

As the PF and CF dimensions are formative, to evaluate the second-order measurement model it is necessary to analyze the multicollinearity of the dimensions, and the value and the statistical significance of the weights. Following the indications of the theory, we use the two-step approach to do so. We therefore work with the scores calculated by the program for each of the first-order components. Table 5 shows the results for the second-order formative measurement model.

All the VIF values are less than five, so there are no problems of multicollinearity [43]. Following the recommendations of Hair et al. [37], which postulate that indicators with non-significant weights should be included if they have loadings above 0.5, only two dimensions did not fulfill these requirements. Nevertheless, both are very close to this value and are statistically significant, so we opt for keeping them [44]. As they are part of a formative scale, removing them would involve the loss of relevant information.

Having concluded the analysis of the measurement model, we analyze the structural model. Firstly, the possible existence of multicollinearity between the antecedent variables of the endogenous construct has been tested. According to Hair et al. [45], there will exist signs of multicollinearity when the VIF indicator is above five. In the structural model proposed problems of multicollinearity do not exist.

After this, a Bootstrapping (5000 samples) was carried out. This provides both the t values and the confidence intervals which allow the evaluation of the statistical significance of the relations. The empirical evidence reveals that there exists a significant relationship between customer factors and perceived impacts and the user intention, so hypotheses H2 and H3 are accepted. Table 6 shows the direct effects included in our research model.

The application of PLS-predict enables evaluating the predictive importance of the constructs. When carrying out procedures, the Q^2^ value is above zero, which backs the predictive relevance for the endogenous construct (Table 6). The coefficient of determination (R^2^) is also examined to evaluate the explanatory power for the endogenous construct and indicates the quantity of variance of a construct, which is explained by the predictive variables of this endogenous construct in the model [46].

Therefore, in the model proposed, the perceived impacts explain 18.25% of the user intention to adopt a sustainable mobility mode of transport and the customer factors 6.34% of it (Table 7).

## 4. Discussion

The data analysis shows various relevant questions. Firstly, the relationship between customer factors (CF) and user intention to adopt a sustainable mobility mode of transport (UI) is confirmed: CF explains 6.34% of the variance of the UI. This indicates that CF positively affects the UI. This finding is in line with the theoretical background [23,24]. Specifically, in the field of mobility, Ahn and Park [2] and Wang et al. [11] advocate the need to carefully consider the user’s perspective in the development of mobility initiatives. In this sense, the implementation of mobility innovations is not a guarantee of their success. Users need to appreciate the benefits they receive from the new transport service and re-place it or combine it with those they used previously. To facilitate success, market orientation suggests that the analysis of consumer perceptions should be a priority when de-signing and launching an innovation, beyond the analysis of product features.

On the other hand, the analysis of the weights of customer forces allows us to hierarchize its formative dimensions for the sector and context analyzed. In this sense, it seems that the existence of previous references is the customer force that gains the most relevance among university students when planning to adopt a sustainable mode of transport. Ranked by relevance, this is followed by the avoidance of physical, economic and social risks, the felt need, and observability. All of them have a direct and positive effect on the formation of CF. This finding faithfully reflects the behavior of the new generations of consumers, who base their buying decisions on the recommendations they read on social networks rather than on the companies’ commercial communication. This new generation of consumers have full access to information about the potential risks of each mode of transport, so they can compare and value the potential loss of money in the case of a wrong purchase; the potential physical damage in case of having an accident; and, also, the potential social risk for using an unfriendly environmental mobility mode of transport. It is also important to note that students are very aware of their felt needs and consequently make their transportation decisions considering what they need in each situation, and are probably less involved with a single mode of transportation than previous generations and tend to adopt mobility as a service.

Secondly, our study reflects that the perceived impacts on the sustainability of the city have a relevant and strong effect on the user’s intention to adopt a sustainable mobility mode of transport, thus also supporting hypothesis H3, which explains 18.25% of the variance. Therefore, if students perceive that adopting a sustainable mobility mode of transport will improve economic development, reduce negative environmental impacts on the city and lead to an improvement in the standard of living of citizens, they will be more inclined to adopt a sustainable mobility mode of transport. This finding is in line with Aguilera et al. [3], who postulate that economic issues or environmental concerns of citizens were drivers of innovation in urban mobility. In this sense, our study is a pioneer in the research on consumer perceptions of the ecological, social and economic impacts of mobility modes of transport and their influence on their adoption.

Thirdly, as we have shown in the previous section, the relationship between product factors and user intentions is not statistically significant, so hypothesis H1 has been rejected. This means that users do not tend to consider product factors when they are considering adopting a new mode of transport. This finding could be controversial, but not under the marketing perspective, which is focused on consumer needs rather than on product design. This market orientation concept has been thoroughly investigated since the early 1990s. All the organization’s members share a series of beliefs which put the customer’s interest first [42] and apply inter-functional resources in a coordinated manner to create higher value [43]. The foundation of this strategic advantage is that the value delivered to the customer is far from being easily duplicated by the firm’s competitors. Following Best [26], “to attain a strong market orientation, a business needs to adopt a market-based management philosophy. The organization restructures itself around markets rather than products or factories, and it develops an employee culture responsive to customers and changing market conditions” [25] (p. 3).

Finally, it is important to consider that the user’s perceived impacts and the customer factor together can only explain 24.59% of the variance. This is an interesting finding as it suggests that there are also other factors that could be considered when modelling users’ intention. As we commented in the theoretical review, the proliferation of research on sustainable mobility, brought about by technological development and the growing concern for sustainability, offers various factors that can affect the intention to use different modes of sustainable transport. Therefore, if we include other factors in our model for future research, we may be able to improve the explanation of UI. Among these factors are compatibility (e.g., Nordhoff et al. [23]; Min et al. [10]; Verma et al. [21] and Wang et al. [20]), attitude towards the use of these transport modes (e.g., Ahn and Park [2]; Eccarius and Lu [24]) or perceived usefulness (e.g., Ahn and Park [2]; Min et al. [10] and Yuen at al. [22]). Other researchers have chosen to incorporate environment-related factors such as environmental concern, values or knowledge (e.g., Ahn and Park [2]; Eccarius and Lu [24] and Verma et al. [21]). Social influence or social norms (e.g., Eccarius and Lu [24]; Min et al. [10] and Nordhoff et al. [23]) and Image in society (e.g., Yuen at al. [22]) have also been studied. Finally, trust (e.g., Nordhoff et al. [23]), performance expectancy (e.g., Nordhoff et al. [23]), awareness-knowledge (e.g., Eccarius and Lu [24]), facilitating conditions (e.g., Nordhoff et al. [23]) or financial incentives (Verma et al. [21]) could be variables to incorporate in the model.

Various limitations prevent the generalization of this study’s results. Firstly, the transversal nature of the research makes it difficult to establish causal relationships. A longitudinal study that assesses the evolution of perceptions and the rate of diffusion could shed light on the relationship analyzed, and let us know if the effect of customer factors and the perceived impact on user intentions to adopt a sustainable mobility mode of transport are really shown in the medium or long term. Secondly, it could also be interesting to analyze the possible mediating effect of the user’s propensity to innovate sustainable mobility modes of transport. Thirdly, all the respondents considered are university students from the city of Seville. The analysis of other people’s perceptions would help us to understand the differences in the effect of customer factors and the perceived impact on user intentions to adopt sustainable mobility that are determined by belonging to another population group. On the other hand, a greater variety of antecedents of user intentions could also be considered. Finally, it is therefore relevant to apply other models that shed light on the forces that condition, and help to explain, the adoption of different sustainable mobility modes of transport from the consumer’s point of view.

## 5. Conclusions

The advantages of developing and implementing sustainable mobility modes of transport are obvious. However, not all sustainable mobility propositions are successfully launched in the market. There is a need to develop tools that help to predict the successful adoption of mobility innovation and in turn help to design that mobility innovation proposition in a way that has a better guarantee of success. Our marketing approach is an innovative point of view that can help us to better understand the reasons for the successful adoption of sustainable mobility modes of transport.

Our model, based on Best [25,26] and Hawkins and Mothersbaugh [27], distinguishes between product forces and customer forces to explain the speed of the adoption of an innovation and thus its diffusion. In the case of sustainable mobility modes of transport, it is the customer forces that have a positive effect on the adoption of the innovation. Consequently, marketing and commercial communication campaigns aimed at citizens should be geared towards reinforcing the positive effects of these customer forces. However, in sustainable mobility, product forces do not seem to have a significant influence on adoption. In this sense, cities and companies that have put their emphasis exclusively on improving mobility products and services, without taking citizens into account, are less likely to succeed. When implementing a new sustainable mobility mode of transport, it is essential to adopt a market orientation. Hence, the perceptions of the citizens must be considered, and the mobility mode of transport must be adapted to the wishes and needs of the users.

In the context of mobility mode of transport, the main customer forces affecting adoption are references, risk avoidance, felt need and observability. Hence, advertising messages and marketing and communication strategies that support the launch of the new mobility proposition should have these four dimensions at their core.

Similarly, the most important product features for users are availability, non-compliance risk and simplicity of use. This indicates that in order to encourage the adoption of the mobility mode of transport, it is necessary to offer wide availability, to fulfill what is promised in the advertisement, avoiding non-compliance risks, and to develop mobility modes of transport which are easier to use.

The adoption of mobility modes of transport is also influenced by the positive or negative impact on the city and its inhabitants. Therefore, it is advisable to plan a mobility model that achieves higher efficiency from the economic point of view and better functionality, higher equity from the social point of view and more protection from the environmental point of view.

Governments must remember that SDG 11 of the 2030 Agenda for Sustainable Development must be the guide for rebuilding their policies to actively improve the lives of all. This means that new public policies and legislation must drive innovation in urban mobility that improves the economic, environmental and social context of cities. The current global situation urges authorities to prioritize sustainable urban development. Hence, in addition to improving and communicating to citizens and tourists the most important consumer adoption forces (i.e., references, risk avoidance, felt need and observability), governments must communicate the beneficial impacts of the new forms of sustainable mobility offered by the city (i.e., economic, environmental and social).

On the other hand, the current diffusion and promising future of shared mobility should not make us ignore safety issues, quite the contrary, as they are necessary to ensure an adequate level of safety and security to avoid injury to users [47]. This way, future lines of research could be related to engineering aspects (i.e., supervised technical condition of the offered vehicles, infrastructure for safe parking of vehicles and lack of additional protection elements for users) and to behavioral aspects (i.e., users’ knowledge in the use of vehicles and respect for rented vehicles and traffic rules). In addition, to improve the quality of the use of these services, we should pay special attention to security, both on the operator’s side (i.e., monitoring and transferring information about user abuse and ensuring high protection of personal data and payment data) and on the society’s side (i.e., reporting any irregularities in IT system use). Specifically, research on security and privacy issues in shared mobility integration is scarce [48].

## Figures and Tables

**Table 1:** Market growth acceleration forces.

Customer Adoption Forces	
Ease of purchase	Existence of few formalities, few difficulties and few people involved in the purchase
Trialability	Facility to have a low-cost (or free) or low-risk trial before purchase
Observability	Ease of observation in use or its positive effects
References	Existence of comments or references on the innovation
Avoid risk	Existence of little or no economic, social or physical risk associated with the trial and use of the innovation
Felt need	The innovation fulfills a recognized need and how strong the need is
Product Adoption Forces	
Affordability	Existence of an acceptable price and/or costs of using the innovation for target customers
Product advantages	Existence of benefits perceived by customer
Simplicity of use	Ease of understanding and use of the innovation. Compatibility with the normal routines of target customers
Availability	Easy access to products at the points of sale preferred by customers
Services	Existence support or maintenance services and other services that increase the value of the innovation
Risk of non-compliance	Existence of little or no performance risk associated with the trial and use of the innovation. Fulfillment of what was promised in the advertisement

**Table 2:** Results of the measurement model.

	Loadings	Cronbach Alpha	Composite Reliability	AVE
**A** **ff** **otdability (AFF)**		0.817	0.891	0.733
PRE1 Affordability of the initial price (for purchase or registration).	0.844			
PRE2 Affordability of the periodic cost (maintenance or recharge).	0.858			
PRE3 Affordability of the total cost of use.	0.866			
**Simplicity of use (SIM)**		0.691	0.861	0.757
FAC1 Easy to use	0.924			
FAC2 Easy to park	0.812			
FAC3 Require a vehicle license to use it *				
**Availability (AVA)**		0.742	0.851	0.659
DIS1 That there are enough vehicles available	0.819			
DIS2 That it is accessible (easy to dispose of)	0.906			
DIS3 That it allows me to move when I need it	0.696			
**Services (SER)**		0.708	0.833	0.630
SER1 Include extras, such as water, air conditioning, helmet…	0.626			
SER2 That I have access to customer service	0.857			
SER3 That it has an app to manage the service	0.874			
**Product advantage (ADV)**		0.737	0.872	0.774
VEN1 That it is fast	0.956			
VEN2 That it is comfortable	0.796			
VEN3 That it allows me to avoid traffic jams *				
**Risk of non compliance (RIS)**		0.720	0.840	0.637
RIN1 That it allows me to move around any area *				
RIN2 That it allows me to move around without timetable restrictions	0.772			
RIN3 That allows me to get to my destination on time	0.832			
RIN4 That it fulfills what was promised in the advertisement	0.790			
**Ease of purchase (PUR)**		0.713	0.837	0.631
UDC1 That it is easy to buy	0.770			
UDC2 That I don’t have to consult with others about my purchase decision	0.770			
UDC3 That it doesn’t require a lot of paperwork to use it	0.842			
**Trialiability (TRI)**		0.858	0.905	0.762
NDP1 That you can try it beforehand	0.843			
NDP2 That you are allowed to try it for free	0.844			
NDP3 That there are demonstrations of its use	0.928			
**Observability (OBS)**		0.835	0.900	0.751
OBS1 That other people around me use it	0.858			
OBS2 That I have seen other people using it.	0.906			
OBS3 That it is a widely used mode of transport	0.835			
**Refferences (REF)**		0.752	0.859	0.672
REC1 That there are positive reviews in my networks	0.874			
REC2 That other people talk positively about its use	0.857			
REC3 That the institutions promote its use	0.720			
**Avoid risk (AVO)**		0.794	0.869	0.628
RIE1 That I avoid the risk of causing an accident	0.876			
RIE2 That it avoids me the risk of having an accident	0.877			
RIE3 That it allows me to control my expenses	0.765			
RIE4 That my family and friends consider a good idea to use it	0.625			
**Felf need (NEC)**		0.886	0.930	0.815
NEC1 That it fits my mobility needs	0.864			
NEC2 That it fits my schedule	0.942			
NEC3 That it fits my frequent destinations	0.901			
**Perceived impacts (PI)**		0.813	0.877	0.643
SOS1 The use of this mode of transport is good for the environment	0.799			
SOS2 The use of this mode of transport is positive for the economy of the city	0.844			
SOS3 The use of this mode of transport helps to reduce social inequalities	0.695			
SOS4 In general, the use of this mode of transport is positive for the city.	0.860			
**User intention (UI)**		0.753	0.843	0.580
IU1 I will use this mode of transport in the future	0.691			
IU2 I will use this mode of transport in the near future	0.585			
IU3 I have positive things to say about this mode of transport	0.864			
IU4 I will recommend this mode of transport to other people	0.867			

* Item eliminated in the refinement process.

**Table 3:** Profile of the respondents.

**Gender**	**Woman** 49.50%	**Man** 50.50%	
**Age**	**Under 21** 33.80%	**21–24** 54.30%	**Over 25** 11.90%
**Driving License**	**Yes** 75.20%	**No** 24.80%	

**Table 4:** Measurement Model. Discriminant Validity. Heterotrait–monotrait ratio (HTMT)—Matrix.

	ADV	AVA	AVO	PI	OBS	AFF	PUR	REF	RIS	SER	USE	NEC	TRI	UI
ADV														
AVA	0.748													
AVO	0.629	0.588												
PI	0.307	0.433	0.410											
OBS	0.290	0.363	0.588	0.222										
AFF	0.210	0.271	0.356	0.273	0.208									
PUR	0.672	0.599	0.731	0.225	0.483	0.346								
REF	0.575	0.470	0.796	0.304	0.726	0.331	0.681							
RIS	0.879	0.758	0.759	0.429	0.369	0.297	0.690	0.655						
SER	0.663	0.542	0.590	0.305	0.316	0.164	0.592	0.669	0.698					
SIM	0.682	0.727	0.564	0.353	0.341	0.287	0.526	0.490	0.552	0.568				
NEC	0.664	0.659	0.703	0.384	0.386	0.383	0.634	0.547	0.850	0.505	0.496			
TRI	0.467	0.357	0.639	0.225	0.343	0.122	0.596	0.611	0.631	0.701	0.340	0.456		
UI	0.179	0.352	0.416	0.605	0.274	0.228	0.237	0.407	0.323	0.311	0.322	0.337	0.229	

**Table 5:** Second-order formative model.

	Vif	Weight	Weight *t*-Value	Loading	Loading *t*-Value
AFF-PF	1.090	0.185	1.065 ^ns^	0.416	2.329 **
SIM-PF	1.589	0.378	1.898 ^ns^	0.691	----------
AVA-PF	1.706	0.418	1.822 ^ns^	0.759	----------
SER-PF	1.415	0.345	1.743 ^ns^	0.672	----------
ADV-PF	1.945	−0.486	2.157 **	----------	----------
RIS-PF	1.959	0.416	1.835 ^ns^	0.688	----------
PUR-CF	1.704	−0.202	0.911 ^ns^	0.489	2.846 **
TRI-CF	1.567	−0.045	0.220 ^ns^	0.515	----------
OBS-CF	1.563	0.064	0.307 ^ns^	0.595	----------
REF-CF	2.112	0.505	1.974 **	----------	----------
AVO-CF	2.274	0.431	1.682 ^ns^	0.857	----------
NEC-CF	1.688	0.382	1.836 ^ns^	0.760	----------

^ns^: not significant ** Sig. at 0.05.

**Table 6:** Results of the structural model.

	Path	*p* Values	T Statistics	Confidence Interval	f^2^	Supported
**PF → UI**	0.123	0.146 ^ns^	1.453	[−0.017; 0.315]	0.012	No
**CF** → **UI**	0.168	0.020 **	2.322	[0.065; 0.347]	0.024	Yes
**PI** → **UI**	0.377	0.000 ***	5.008	[0.217; 0509]	0.167	Yes

*** *p* < 0.001, ** *p* < 0.01, ^ns^: not significant t (0.05; 4999) = 1.64791345; t (0.01; 4999) = 2.333843952; t (0.001; 4999) = 3.106644601 Sig. at 0.05.

**Table 7:** Effects on the endogenous variables.

	R^2^	Q^2^	Direct Effect	Correlations	Variance Explained
User Intention (UI)	0.292	0.217			
H1: Product Factors (PF)			0.123	0.375	4.61%
H2: Customer Factors (CF)			0.168	0.376	6.34%
H3: Perceived Impacts (PI)			0.377	0.484	18.25%

## Data Availability

Not applicable.

## References

[1] Chen S.Y., Lu C.C. (2016). Exploring the Relationships of Green Perceived Value, the Diffusion of Innovations, and the Technology Acceptance Model of Green Transportation. Transp. J..

[2] Ahn H., Park E. (2022). For Sustainable Development in the Transportation Sector: Determinants of Acceptance of Sustainable Transportation Using the Innovation Diffusion Theory and Technology Acceptance Model. Sustain. Dev..

[3] Aguilera-García Á., Gomez J., Sobrino N. (2020). Exploring the Adoption of Moped Scooter-Sharing Systems in Spanish Urban Areas. Cities.

[4] Munkácsy A., Monzón A. (2018). Diffusion of Bike Sharing as an Innovation Vector in the City: The Case of BiciMAD (Madrid). J. Urban Technol..

[5] Medina-Molina C., Pérez-Macías N. (2022). The Identification of Causal Mechanisms in Sustainable Urban Transitions—A Systematic Approach to Case Selection. Mathematics.

[6] Luque-Vega L.F., Carlos-Mancilla M.A., Payán-Quiñónez V.G., Lopez-Neri E. (2020). Smart Cities Oriented Project Planning and Evaluation Methodology Driven by Citizen Perception-IoT Smart Mobility Case. Sustainability.

[7] Ahmed W., Hizam S.M., Sentosa I. (2022). Reviewing the Components of Technology Acceptance Behavior in Transportation Sector. Commun. Sci. Lett. Univ. Žilina.

[8] Böcker L., Anderson E., Uteng T.P., Throndsen T. (2020). Bike Sharing Use in Conjunction to Public Transport: Exploring Spatiotemporal, Age and Gender Dimensions in Oslo, Norway. Transp. Res. Part A Policy Pract..

[9] Ho C.Q., Mulley C., Hensher D.A. (2020). Public Preferences for Mobility as a Service: Insights from Stated Preference Surveys. Transp. Res. Part A Policy Pract..

[10] Min S., So K.K.F., Jeong M. (2019). Consumer Adoption of the Uber Mobile Application: Insights from Diffusion of Innovation Theory and Technology Acceptance Model. J. Travel Tour. Mark..

[11] Wang X., Yan X., Zhao X., Cao Z. (2022). Identifying Latent Shared Mobility Preference Segments in Low-Income Communities: Ride-Hailing, Fixed-Route Bus, and Mobility-on-Demand Transit. Travel Behav. Soc..

[12] Goldman T., Gorham R. (2006). Sustainable Urban Transport: Four Innovative Directions. Technol. Soc..

[13] Bąk A., Nawrocka E., Jaremen D.E. (2022). “Sustainability” as a Motive for Choosing Shared-Mobility Services: The Case of Polish Consumers of Uber Services. Sustainability.

[14] Roger E. (1995). Diffusion of Innovations.

[15] Wang Y.Y., Wang Y.S., Lin H.H., Tsai T.H. (2019). Developing and Validating a Model for Assessing Paid Mobile Learning App Success. Interact. Learn. Environ..

[16] Moore G.C., Benbasat I. (1991). Development of an Instrument to Measure the Perceptions of Adopting an Information Technology Innovation. Inf. Syst. Res..

[17] Davis F.D. (1989). Perceived Usefulness, Perceived Ease of Use, and User Acceptance of Information Technology. MIS Q. Manag. Inf. Syst..

[18] Ajzen I. (1991). The Theory of Planned Behavior. Organ. Behav. Hum. Decis. Process..

[19] Venkatesh V., Morris M.G., Davis G.B., Davis F.D. (2003). User Acceptance of Information Technology: Toward a Unified View. MIS Q..

[20] Wang Y., Douglas M., Hazen B. (2021). Diffusion of Public Bicycle Systems: Investigating Influences of Users’ Perceived Risk and Switching Intention. Transp. Res. Part A Policy Pract..

[21] Verma M., Verma A., Khan M. (2020). Factors Influencing the Adoption of Electric Vehicles in Bengaluru. Transp. Dev. Econ..

[22] Yuen K.F., Cai L., Qi G., Wang X. (2021). Factors Influencing Autonomous Vehicle Adoption: An Application of the Technology Acceptance Model and Innovation Diffusion Theory. Technol. Anal. Strateg. Manag..

[23] Nordhoff S., Malmsten V., van Arem B., Liu P., Happee R. (2021). A Structural Equation Modeling Approach for the Acceptance of Driverless Automated Shuttles Based on Constructs from the Unified Theory of Acceptance and Use of Technology and the Diffusion of Innovation Theory. Transp. Res. Part F Traffic Psychol. Behav..

[24] Eccarius T., Lu C.C. (2020). Adoption Intentions for Micro-Mobility—Insights from Electric Scooter Sharing in Taiwan. Transp. Res. Part D Transp. Environ..

[25] Best R.J. (2007). Marketing Estratégico.

[26] Best R.J. (2013). Market-Based Management: Strategies for Growing Customer Value and Profitability.

[27] Hawkins D.I., Mothersbaugh D.L. (2010). Consumer Behaviour: Building Marketing Strategies.

[28] Plan de Movilidad Urbana Sostenible Del Municipio de Sevilla. Diagnóstico 2019. https://www.sevilla.org/servicios/movilidad/pmus.

[29] Tsavachidis M., Petit Y. (2022). Le Re-Shaping Urban Mobility—Key to Europe’s Green Transition. J. Urban Mobil..

[30] del Río-Vázquez M.-E.S., Rodríguez-Rad C.J., Revilla-Camacho M.-Á. (2019). Relevance of Social, Economic, and Environmental Impacts on Residents’ Satisfaction with the Public Administration of Tourism. Sustainability.

[31] Wu X., Zhi Q. (2016). Impact of Shared Economy on Urban Sustainability: From the Perspective of Social, Economic, and Environmental Sustainability. Energy Procedia.

[32] Deakin E. (2001). Sustainable Development and Sustainable Transportation: Strategies for Economic Prosperity, Environmental Quality, and Equity. Sustain. Dev..

[33] Büyüközkan G., Feyzioğlu O., Göçer F. (2018). Selection of Sustainable Urban Transportation Alternatives Using an Integrated Intuitionistic Fuzzy Choquet Integral Approach. Transp. Res. Part D Transp. Environ..

[34] Gallo M., Marinelli M. (2020). Sustainable Mobility: A Review of Possible Actions and Policies. Sustain..

[35] Kopplin C.S., Brand B.M., Reichenberger Y. (2021). Consumer Acceptance of Shared E-Scooters for Urban and Short-Distance Mobility. Transp. Res. Part D Transp. Environ..

[36] Reibstein D.J. (1978). The Prediction of Individual Probabilities of Brand Choice. J. Consum. Res..

[37] Hair J.F., Hult G.T., Ringle C.M., Sarstedt M., Castz J., Cepeda Carrión G., Roldán J.L. (2019). Manual de Partial Least Squares Structural Equation Modeling (Pls-Sem).

[38] Statistics I.B.M.S. (2019). Released IBM SPSS Statistics for Windows.

[39] Ringle C.M., Wende S., Becker J.-M. (2015). SmartPLS 3.

[40] Hair J.F., Risher J.J., Sarstedt M., Ringle C.M. (2019). When to Use and How to Report the Results of PLS-SEM. Eur. Bus. Rev..

[41] Hair Jr J.F., Sarstedt M., Ringle C.M., Gudergan S.P. (2017). Advanced Issues in Partial Least Squares Structural Equation Modeling.

[42] Ray S.K., Sahney S. (2021). Personal Cultural Orientation and Green Purchase Intention: A Case of Electric Two-Wheelers in India. J. Asia Bus. Stud..

[43] Wong K.K.-K. (2019). Mastering Partial Least Squares Structural Equation Modeling (PLS-Sem) with Smartpls in 38 Hours.

[44] Roberts N., Thatcher J. (2009). Conceptualizing and Testing Formative Constructs: Tutorial and Annotated Example. ACM Sigmis Database Database Adv. Inf. Syst..

[45] Hair J.F., Henseler J., Dijkstra T., Sarstedt M., Ringle C., Diamantopoulos A., Straub D., Ketchen D., Gtm H., Calantone R. (2014). Common Beliefs and Reality about Partial Least Squares: Comments on Rönkkö and Evermann. Organ. Res. Methods.

[46] Chin W.W. (2010). How to Write up and Report PLS Analyses. Handbook of Partial Least Squares.

[47] Turoń K., Czech P., Tóth J. (2019). Safety and Security Aspects in Shared Mobility Systems. Sci. J. Sil. Univ. Technol. Ser. Transp..

[48] Affia A.A.O., Matulevicius R. Security Risk Management in Shared Mobility Integration. Proceedings of the 17th International Conference on Availability, Reliability and Security (ARES ‘22).

